# Draft Genome Sequence of Tenacibaculum ovolyticum To-7Br, Recovered from a Farmed Atlantic Salmon (*Salmo salar*)

**DOI:** 10.1128/mra.00254-22

**Published:** 2022-06-02

**Authors:** Ruben Avendaño-Herrera, Mónica Saldarriaga-Córdoba, Rute Irgang

**Affiliations:** a Universidad Andrés Bello, Laboratorio de Patología de Organismos Acuáticos y Biotecnología Acuícola, Facultad de Ciencias de la Vida, Viña del Mar, Chile; b Centro FONDAP, Interdisciplinary Center for Aquaculture Research, Universidad Andrés Bello, Viña del Mar, Chile; c Centro de Investigación Marina Quintay, Universidad Andrés Bello, Valparaíso, Chile; d Centro de Investigación en Recursos Naturales y Sustentabilidad, Universidad Bernardo O'Higgins, Santiago, Chile; Indiana University, Bloomington

## Abstract

We present the draft genome sequence of Tenacibaculum ovolyticum isolate To-7Br, recovered from a gill of a farmed specimen of Atlantic salmon (Salmo salar) showing signs of tenacibaculosis. This study provides the first detailed insights into the genomic characteristics of *T. ovolyticum* isolated for the first time from fish farmed in Chile.

## ANNOUNCEMENT

Species of the genus *Tenacibaculum* are Gram-negative, filamentous bacteria with gliding motility and are commonly found in marine environments (1). To date, 31 *Tenacibaculum* species are validly described (https://www.bacterio.net/), including several microorganisms recognized as putative pathogens associated with tenacibaculosis in fish (2, 3). Studies on the bacterial microbiota associated with fish diseases have described the presence of Tenacibaculum dicentrarchi (4), Tenacibaculum finnmarkense (5), Tenacibaculum maritimum (6, 7), and Tenacibaculum piscium (8) in Chilean salmon aquaculture. The present study describes the presence and genome sequence of a *T. ovolyticum* isolate retrieved from a gill of Atlantic salmon (Salmo salar) farmed in southern Chile (Seno Gala, 44°11′21″S, 73°8′1″W) in April 2018.

Strain To-7Br was obtained during a tenacibaculosis outbreak, with isolation on a marine agar 2216 (BD Difco) plate incubated at 18°C for 48 h, which resulted in mixed cultures. The dominant colony morphotype was streaked onto new marine agar 2216 plates to obtain pure cultures, which were stored at −80°C in marine broth supplemented with 10% (vol/vol) glycerol. The taxonomic position of the organism was first determined by 16S rRNA gene sequencing by Macrogen. Two pure colonies were subjected to DNA extraction by employing the InstaGene matrix (Bio-Rad) according to the manufacturer’s instructions. The 16S rRNA gene was PCR amplified using the universal primer pair pA and pH (9). The full-length 16S rRNA gene sequence (1,341 nucleotides) with the accession number OM420283 revealed 99.87% identity to *T. ovolyticum* strain da5A-8 (LC144619) and 99.85% identity to the type strain, IFO 15947 (NR_040912).

For genomic sequencing, DNA was purified with the Mag-Bind universal pathogen 96 kit (Omega Bio-Tek Inc.), and concentration was measured using the QuantiFluor dsDNA system on a Quantus fluorometer (Promega). Prior to *de novo* genome assembly, raw Illumina sequence data were quality checked with FastQC v.0.11.9 (https://www.bioinformatics.babraham.ac.uk/projects/fastqc/). Next-generation sequencing reads were preprocessed with the Geneious Prime v.2020.2.2 software (10), using the following workflow: (i) reads were paired using the “set paired reads” function (insert size = 300 bp); (ii) poor-quality bases were trimmed from read ends using the BBDuk Trimmer plugin (reads of <20 bp and those with a minimum quality score of <30 were removed), with paired-read overlaps trimmed to ensure complete adapter removal; (iii) coverage was normalized by down-sampling reads in high-depth genome areas of the genome with the “error correct” and “normalize reads” functions using BBNorm; and (d) duplicate reads were removed using the Dedupe plugin. Assembly was performed with SPAdes v.3.15.2 run in Geneious Prime, and quality was checked by QUAST version 5.1 (11). This generated 97 contigs and 4,054,048 bp with a G+C content of 29.58%. To quantitatively evaluate the assembly quality, the following measures were considered: minimum (i.e., 1,026 bp), maximum (i.e., 323,162 bp), and average (i.e., 41,794 bp) contig length. The total assembly size coincided with the expected size of the genome (12). The draft genome sequence contained 3,706 coding sequences, 244 subsystems, and 46 RNAs (42 tRNAs, 3 pseudo-tRNAs, and 1 16S rRNA), as determined by using the RAST server v.2.0 (13, 14).

The *in silico* genome analysis revealed genes related to secretion systems, including the type IV secretion system (T4SS), T1SS, T6SS, and T9SS, as detected by TXSScan (http://mobyle.pasteur.fr/cgi-bin/portal.py#forms::txsscan). In addition, iron-related protein families involved in iron transport, siderophore synthesis, siderophore transport, transcriptional regulation, and iron storage were identified. Furthermore, hemolysis genes and genes associated with antibiotic resistance (i.e., resistance to tetracycline and fluoroquinolones), the stress response, and other metabolic pathways were found. The present study provides the first detailed insights into the genomic characteristics of *T. ovolyticum* isolated for the first time from fish farmed in Chile. Considering the culture viability of isolate To-7Br, more details about virulence potential will be reported in a future publication.

### Data availability.

This whole-genome shotgun project has been deposited at DDBJ/ENA/GenBank under the accession number JAKLCZ000000000.1 (https://www.ncbi.nlm.nih.gov/nuccore/JAKLCZ000000000). The version described in this paper is version JAKLCZ010000000. BioProject ID PRJNA801316 is publicly available at https://www.ncbi.nlm.nih.gov/bioproject/PRJNA801316, containing BioSample SAMN25339059 (https://www.ncbi.nlm.nih.gov/biosample/SAMN25339059). The GenBank accession number for the 16SrRNA gene sequence is OM420283.
